# Systemic Lupus Erythematosus: Perinatal Outcomes in Patients Treated With and Without Hydroxychloroquine

**DOI:** 10.31486/toj.20.0013

**Published:** 2020

**Authors:** Sima Baalbaki, Jeff M. Szychowski, Ying Tang, Luisa Wetta, Akila Subramaniam

**Affiliations:** ^1^ Department of Obstetrics and Gynecology and Women's Health, Montefiore Medical Center/Albert Einstein College of Medicine, Bronx, NY; ^2^ Department of Obstetrics and Gynecology, Maternal-Fetal Medicine Division, Center for Women's Reproductive Health, School of Medicine at the University of Alabama at Birmingham, Birmingham, AL

**Keywords:** *Hydroxychloroquine*, *lupus erythematosus–systemic*, *pregnancy*

## Abstract

**Background:** Systemic lupus erythematosus (SLE) is an autoimmune disease commonly encountered during pregnancy. The use of hydroxychloroquine (HCQ) for SLE treatment in pregnancy has been supported by a few small studies performed in populations dissimilar from populations in the United States. Our objective was to compare maternal and neonatal outcomes in pregnant patients with SLE treated with and without HCQ at a tertiary care center in the United States.

**Methods:** We conducted a retrospective cohort study of patients with SLE and singleton gestations who delivered at the University of Alabama at Birmingham from 2006 to 2013. Patients treated with HCQ during pregnancy were compared with patients who did not receive HCQ. Key outcomes included maternal morbidities (hypertensive disorders, intrauterine growth restriction, preterm delivery, venous thromboembolism), disease-related morbidity, maternal death, and a composite of neonatal morbidity. Outcomes were compared using chi-square, Fisher exact, Wilcoxon rank sum, and *t* tests. Odds of adverse outcomes were modeled with logistic regression.

**Results:** Seventy-seven patients with SLE were included for analysis; 47 (61%) were treated with HCQ and 30 (39%) were not. We found no differences in the rates of maternal morbidities or death between groups. Patients taking HCQ had increased rates of disease-related hospitalizations (43% vs 7%, *P*<0.01) and inpatient rheumatology consultations (38% vs 10%, *P*<0.01), increases that persisted after multivariable adjustments (adjusted odds ratio [aOR] 8.09, 95% confidence interval [CI] 1.60-40.9; aOR 4.50, 95% CI 1.08-18.6, respectively). Neonatal morbidity did not differ between groups.

**Conclusion:** We found no differences in major maternal or neonatal outcomes in pregnant patients with SLE managed with HCQ.

## INTRODUCTION

Systemic lupus erythematosus (SLE) is one of the most common autoimmune diseases encountered during pregnancy, with approximately 4,500 women with SLE becoming pregnant annually.^1-2^ Women with SLE are at increased risk of adverse pregnancy outcomes. Poor maternal outcomes include disease flares, worsening of disease, hypertensive complications of pregnancy, and renal disease.^1-3^ SLE-associated fetal and neonatal risks include miscarriage, stillbirth, preterm delivery, intrauterine growth restriction, neonatal cardiac disease, and neonatal death.^1,3^

Although a considerable amount of literature focuses on these adverse pregnancy outcomes, few studies describe the optimal therapeutic management of SLE during pregnancy. Many medications routinely used in the treatment of SLE in nonpregnant patients, including mycophenolate, methotrexate, and cyclophosphamide, are contraindicated during pregnancy.^2^ Hydroxychloroquine (HCQ) is one treatment option that appears to be safe in pregnancy and has been shown to reduce the risk of disease flares and maternal mortality.^2,4,5-9^ Continuing the use of HCQ, even during the first trimester, has not been associated with an increased risk for fetal anomalies.^1,2,5-9^ As such, both the British Society for Rheumatology and the European League Against Rheumatism have recommended HCQ for the treatment of SLE during pregnancy.^10,11^

Use of HCQ is also supported by a few small studies evaluating the impact of HCQ on preterm delivery, birthweight, growth restriction, and hypertensive disorders of pregnancy.^4,9,12-15^ However, the majority of the studies evaluating maternal and neonatal outcomes have been performed in populations dissimilar to the racial and ethnic makeup of the United States, making the generalizability of these studies to the US population difficult. No guidelines for the use of HCQ in pregnancy have been specifically endorsed in the United States. Our objective was to compare the maternal and neonatal outcomes of pregnant patients with SLE based on treatment with or without HCQ in a large tertiary referral center in the southern United States. We hypothesized that HCQ use in pregnancy would not increase maternal or neonatal morbidities often encountered in patients with SLE.

## METHODS

After institutional review board approval (#X130423012), we identified all patients with SLE and a singleton gestation who delivered at our institution from 2006 to 2013. All included patients had a rheumatologist-confirmed prepregnancy diagnosis of SLE. Patients with multifetal gestations and prenatally diagnosed congenital anomalies were excluded. Fetal anomalies were excluded because the focus of this study was on maternal and neonatal outcomes, not safety or teratogenicity of HCQ, as safety has previously been evaluated.^12,13,16,17^ In addition, patients with confirmed antiphospholipid syndrome and on anticoagulation were also excluded. If a patient had more than one pregnancy during the study period, only the first pregnancy was included for analysis.

In this retrospective cohort study, patients with SLE who were treated with HCQ during pregnancy were compared with patients who did not receive HCQ. Use of HCQ was determined by the rheumatologist managing the patient's SLE. Individual maternal and neonatal medical records were reviewed, and data were abstracted and entered into a constructed database (Access 2010; Microsoft Corp). Patient demographics (age, race, parity, and body mass index [BMI]) and maternal medical comorbidities were evaluated as possible covariates. Delivery characteristics such as gestational age at delivery, mode of delivery, and antepartum and postpartum complications were identified. In addition, disease-related characteristics such as lupus classification; positive antibodies, including Sjögren syndrome type A (SSA) and Sjögren syndrome type B (SSB); duration of disease; last flare; duration of medication use; and lupus-associated morbidities (eg, nephritis, pneumonitis, and cerebritis) were collected. An important note is that while disease-related characteristics were abstracted from the patients’ medical records, the routine evaluation of baseline disease status during the first prenatal visit at our institution (to evaluate perinatal risk during pregnancy) includes assessment of renal function (serum creatinine, urine protein/creatinine ratio, or 24-hour collected urine protein), blood pressure, and hematologic parameters (complete blood count). These laboratory values were used as a marker for disease activity related to obstetric outcomes. Because not all patients had a rheumatologic evaluation within 6 weeks of their initial visit, the validity of their self-reported disease status was hard to evaluate. For this reason, the collected laboratory values were used as surrogate markers.

Key maternal outcomes included hypertensive disorders in pregnancy (gestational hypertension; preeclampsia; eclampsia; and hemolysis, elevated liver enzymes, and low platelets [HELLP] syndrome); intrauterine growth restriction; preterm birth; infectious complications; venous thromboembolism; and maternal death. Disease-specific maternal outcomes, including inpatient consultation by rheumatology or disease-related hospitalizations in pregnancy, were also assessed. Neonatal outcomes included fetal death, neonatal death prior to infant discharge, and a composite of neonatal morbidity and mortality. The neonatal composite morbidity included neonatal intensive care unit admission >72 hours, mechanical ventilation >24 hours, suspected sepsis, culture-proven early-onset sepsis (first 7 days of life), grade III/IV intraventricular hemorrhage, hypoxic ischemic encephalopathy, respiratory distress syndrome, seizures, requirement for cardiopulmonary resuscitation, and necrotizing enterocolitis. Individual components of the neonatal composite were analyzed independently. We considered the neonatal diagnosis to have been made if the diagnosis was included in the infant's discharge summary and documented by neonatologists.

Outcomes were directly compared between patients treated with HCQ during pregnancy and patients who did not receive HCQ. The *t* test or Wilcoxon rank sum test was used for analysis of continuous variables, while categorical variables were analyzed with chi-square test or Fisher exact test as appropriate. Multivariable logistic regression controlling for covariates was used to calculate adjusted odds ratios (aORs) with 95% confidence intervals (CIs) for adverse outcomes. All statistical analysis was conducted using SAS, version 9.2 (SAS Institute Inc). Results were considered statistically significant if *P* was <0.05. No adjustments were made for multiple comparisons.

## RESULTS

A total of 77 females with SLE were included in the analysis; 47 (61%) were treated with HCQ during pregnancy and 30 (39%) were not. Baseline demographics and other characteristics of the 2 groups are presented in Table 1. Patients treated with HCQ were more likely to be African American, younger, and nulliparous. Patients treated with HCQ were also more likely to be concomitantly treated with oral corticosteroids during their pregnancy (83% vs 40%, *P*<0.01). Mean BMI, rates of chronic hypertension, and rates of diabetes mellitus were not significantly different between the 2 groups. The groups also had similar baseline serum creatinine, blood pressure, and proteinuria measurements. Although the data are not presented, these parameters were similar both at the prepregnancy assessment and at the time of delivery.

**Table 1.** Patient Demographics and Baseline Characteristics

Variable	Hydroxychloroquine Group, n=47	No Hydroxychloroquine Group, n=30	*P* value
Maternal age, years, mean ± SD	26.2 ± 5.2	30.5 ± 5.8	<0.01
Nulliparous	28 (60)	10 (33)	0.03
Race			
White	10 (21)	15 (50)	
Black	36 (77)	14 (47)	0.01
Other	1 (2)	1 (3)	
Body mass index, kg/m^2^, mean ± SD	29.0 ± 7.6	29.0 ± 6.9	0.97
Tobacco use	4 (9)	4 (13)	0.70
Alcohol use	0 (0)	0 (0)	–
Drug use	1 (2)	1 (3)	>0.99
Payment source			
Private	13 (33)	10 (36)	
Medicaid	27 (68)	15 (54)	0.10
Uninsured/self-pay	0 (0)	3 (11)	
Marital status			
Single	34 (72)	17 (57)	0.16
Married	13 (28)	13 (43)	
Chronic hypertension	15 (32)	9 (30)	0.86
Received ongoing rheumatologic care	43 (91)	21 (70)	0.01
Diabetes mellitus	6 (13)	6 (20)	0.52
Gestational diabetes	2 (4)	3 (10)	0.37
Pregestational diabetes	4 (8)	3 (10)	>0.99
Prior cesarean delivery	7 (15)	3 (10)	0.73
Baseline serum creatinine, mg/dL, mean ± SD	0.75 ± 0.80^a^	0.80 ± 0.58	0.76
Baseline systolic blood pressure, mmHg, mean ± SD	115.7 ± 16.1	118.3 ± 17.5	0.51
Baseline diastolic blood pressure, mmHg, mean ± SD	68.3 ± 9.8	70.4 ± 16.5	0.54
Baseline proteinuria (protein/creatinine ratio), mean ± SD	0.72 ± 1.59^b^	0.42 ± 0.91^b^	0.34
Delivery route			
Vaginal	28 (60)	14 (47)	0.27
Cesarean	19 (40)	16 (53)	
Prednisone use during pregnancy^c^	39 (83)	12 (40)	<0.01
Aspirin use during pregnancy^d^	33 (70)	20 (67)	0.74

Note: Data are presented as n (%) unless otherwise indicated.

^a^No negative values for baseline serum creatinine were noted. However, 0 was recorded for some baseline values.

^b^No negative values for baseline proteinuria were noted. However, 0 was recorded for some baseline values.

^c^Includes all patients prescribed prednisone for management of systemic lupus erythematosus anytime during pregnancy.

^d^Includes all patients prescribed low-dose daily aspirin for prophylaxis during pregnancy.

Unadjusted results for maternal outcomes are presented in Table 2. No significant differences in the rate of maternal mortality or in any of the maternal morbidity measures were found between the treatment groups. However, patients treated with HCQ were more likely to require inpatient hospitalization for disease-related complications than patients not treated with HCQ (43% vs 7%, *P*<0.001; odds ratio (OR) 10.4, 95% CI 2.21-48.7). Furthermore, patients treated with HCQ were more likely to require inpatient consultation by rheumatology than patients not treated with HCQ (38% vs 10%, *P*=0.007; OR 5.59, 95% CI 1.48-21.1). The increase in disease-related hospitalization and inpatient rheumatology consultation persisted even after multivariable adjustments for age and parity (aOR 8.09, 95% CI 1.60-40.9; aOR 4.50, 95% CI 1.08-18.6, respectively). While the patients treated with HCQ had more hospitalizations and rheumatology consultations than the patients not treated with HCQ , the need for intensive care unit admission did not differ between the 2 groups (3 patients [6%] in the HCQ group vs 1 patient [3%] in the no HCQ group, *P*>0.99).

**Table 2.** Maternal and Neonatal Outcomes

Variable	Hydroxychloroquine Group, n=47	No Hydroxychloroquine Group, n=30	*P* Value
Hypertensive disorders in pregnancy^a^	14 (30)	8 (27)	0.77
Preeclampsia with severe features	9 (19)	7 (23)	0.66
Intrauterine growth restriction	1 (2)	3 (10)	0.29
Gestational weeks at delivery, mean ± SD	35.5 ± 4.9	35.6 ± 4.2	0.88
Preterm birth <37 weeks	19 (40)	16 (53)	0.27
Infectious complications	8 (17)^b^	5 (17)	0.97
Chorioamnionitis	5 (11)	2 (7)	0.70
Endometritis	4 (9)	3 (10)	>0.99
Venous thromboembolism	2 (4)	0 (0)	0.52
Maternal death	2 (4)	1 (3)	>0.99
Inpatient consultation for rheumatology	18 (38)	3 (10)	0.007
Median number of consults (Q_1_-Q_3_)	1 (1-1)	2 (1-3)	
Disease-related hospitalization during pregnancy	20 (43)	2 (7)	<0.001
Neonatal composite^c^	18 (38)	15 (50)	0.31
Intrapartum fetal death	2 (4)	0 (0)	0.52
NICU admission >72 h	16 (34)	15 (50)	0.16
Mechanical ventilation >24 h	2 (4)	5 (17)	0.10
Suspected sepsis	5 (11)	1 (3)	0.40
Culture-proven early-onset sepsis	2 (4)	0 (0)	0.52
Neonatal death	1 (2)	1 (3)	>0.99
Grade III/IV intraventricular	0 (0)	0 (0)	–
hemorrhage			
Hypoxic ischemic encephalopathy	1 (2)	0 (0)	>0.99
Respiratory distress syndrome	4 (9)	4 (13)	0.70
Seizures	0 (0)	0 (0)	–
Cardiopulmonary resuscitation	1 (2)	0 (0)	>0.99
Necrotizing enterocolitis	2 (4)	0 (0)	0.52

Notes: Data are presented as n (%) unless otherwise indicated. Because no maternal pregnancy or neonatal morbidities were statistically significant, no odds ratios or adjustments for age and parity are presented. The adjusted odds ratio of maternal disease-related morbidity is presented in the body of the text.

NICU, neonatal intensive care unit.

^a^Hypertensive disorders in pregnancy denotes any diagnosis of gestational hypertension or preeclampsia.

^b^One patient was treated for both chorioamnionitis and endometritis.

^c^Neonatal composite includes the 12 listed outcomes.

No differences were found between groups in the unadjusted results for neonatal composite morbidity or the individual components of the composite, including neonatal birthweight (data not shown). Neonatal death was similar in the 2 groups (1 neonatal death in each group, *P*>0.99). No heart block was noted in neonates born to SSA- or SSB-positive mothers.

## DISCUSSION

Our study suggests that HCQ does not have a significant effect—either positive or negative—on major maternal and neonatal outcomes in a predominantly African American population treated in a large tertiary center in the southern United States. However, the results suggest a relationship between the use of HCQ and an increased number of disease-related hospitalizations and inpatient consultations by rheumatology. These results may be attributable to the unfamiliarity of obstetricians with HCQ instead of a more severe baseline disease status in patients being treated with HCQ, as no differences were detected in rates of chronic hypertension, diabetes, renal dysfunction, and baseline blood pressure between patients on HCQ and patients who were not treated with HCQ. Therefore, although patients do not seem to be at any increased risk for adverse pregnancy outcomes because of HCQ use, they may require closer rheumatologic follow-up during pregnancy than patients not treated with HCQ.

Patients with SLE are at higher risk for adverse complications during pregnancy compared to patients without the disease. A 2-year population-based study of 555 pregnant patients with SLE and approximately 600,000 pregnant controls demonstrated a statistically significant increase in the incidence of hypertensive complications, nonelective cesarean deliveries, preterm delivery, and pregnancy-related thromboses in the patients with SLE compared to the control group.^3^ Among pregnant patients with SLE, hypertensive disorder rates of 20% to 30%, preeclampsia rates of 13% to 35%, and preterm delivery rates of 20% to 54% have been reported.^2,3^ Our rates for these outcomes were comparable, suggesting that our cohort was representative of previous study populations from this perspective. However, unlike our study, prior studies did not specifically assess rates of these outcomes for a specific treatment.

Because disease quiescence prior to conception and throughout pregnancy correlates with more favorable pregnancy outcomes, identifying a therapy that is safe for use during pregnancy is important.^18^ The antimalarial medication HCQ has been used for years as a therapy for individuals with SLE.^12,13^ Several studies have shown that HCQ appears to be safe for both the mother and fetus during pregnancy.^2,4-9,12,17^ Most studies have focused principally on fetal outcomes such as congenital anomalies, spontaneous abortion, fetal death, and preterm birth.^4,12,13,17^ Clowse et al evaluated the effect of discontinuing HCQ treatment immediately prior to conception or during the first trimester compared to continuing therapy throughout pregnancy.^16^ Higher disease activity was noted in patients who discontinued HCQ compared to patients who continued the medication, consistent with other reports suggesting that continuation of HCQ during pregnancy decreases the risk of disease flares.^2,4-8,17^ Some small studies have evaluated specific obstetric and neonatal outcomes associated with HCQ use in pregnancy. Izmirly et al showed that HCQ use during pregnancy was associated with a reduced risk of developing the cardiac manifestations of neonatal lupus in fetuses born to mothers with anti-Ro/SSA or anti-La/SSB antibodies.^19^ In a prospective study of 20 patients, Levy et al noted higher mean gestational ages at delivery and higher Apgar scores for babies of patients treated with HCQ vs patients treated without HCQ (prednisone alone).^4^ In contrast, a larger study (n=114) by Dav-Citrin et al noted that patients treated with HCQ during pregnancy delivered at an earlier gestational age, had higher rates of preterm birth, and had babies with lower birthweights than patients who were not treated with HCQ.^12^ A proposed mechanism for these disparate findings was the potential of greater disease burden in patients treated with HCQ. However, in a systematic review (n=631), Sperber et al found no difference in the rate of preterm birth between patients treated with HCQ and patients not treated with HCQ.^13^ While these studies had a larger number of cases compared to our study, specific pregnancy outcomes such as hypertensive disease and intrauterine growth restriction were not specifically examined. In one of the few studies that evaluated obstetric outcomes in patients taking HCQ, Buchanan et al noted no differences in rates of prematurity, intrauterine growth restriction, neonatal birthweight, maternal hypertension, or preeclampsia in the 36 patients studied.^9^ A 2015 study by Leroux et al (n=118) showed a decreased rate of intrauterine growth restriction and preterm delivery associated with HCQ use.^14^ While Leroux et al also evaluated other obstetric outcomes, their patient population was different from our cohort, as their population was primarily Caucasian with a lower BMI and greater maternal age. Thus, unlike the primarily European studies previously done, our study contributes an analysis of specific maternal and neonatal morbidities that have not previously been evaluated in a population that is predominantly African American, is covered by government insurance, and has a greater BMI at a large tertiary care referral center in the southern United States. Moreover, although our study shows no improvement in maternal or neonatal outcomes based on HCQ use, equally as important is the absence of an increase in deleterious effects among patients treated with HCQ.

One strength of this study is that it was conducted at a large tertiary center caring for a large population of high-risk patients with access to rheumatologic care and consultation services. In addition, the study cohort is well described, with similar demographics and baseline characteristics between treatment groups. Furthermore, this cohort is much different from populations previously studied, as ours was largely an indigent, African American population with higher BMIs, making our cohort more applicable to the racial and physical makeup of the southern United States. In addition, our baseline rates of preterm birth and hypertensive disorders of pregnancy are consistent with rates documented in the literature.

Limitations of this study include the small sample size because of the rare nature of the disease, with power limited to detect only large clinically meaningful and statistically significant group differences. In addition, because the HCQ group was associated with greater disease-related morbidity, we acknowledge the difficulty in attaining specific measures to assess baseline disease activity in the patients. In other words, patients treated with HCQ may have had more severe baseline disease, possibly accounting for some of the more frequent hospitalizations and consultations by rheumatology and potentially explaining the lack of decrease in rates of maternal complications in patients taking HCQ vs patients not. However, given the similarity in our groups in assessment of baseline health status routinely measured at prenatal visits (chronic hypertension, diabetes, renal dysfunction, and baseline blood pressure), we cannot say with certainty that the HCQ group had more severe disease that would be related to obstetric outcomes. Another limitation is the difference in prednisone use among the groups. We acknowledge as a limitation the inability to account for total prednisone usage and the decreased dosage possibly afforded by HCQ use. Further, the same rheumatologist did not manage all the patients in the study, potentially resulting in different disease management practices in our cohort.

## CONCLUSION

This study suggests that HCQ use in pregnancy does not increase or decrease obstetric or neonatal morbidities often encountered in patients with SLE. However, patients treated with HCQ during pregnancy may require closer rheumatologic follow-up and increased inpatient care than their similarly matched counterparts who do not take HCQ during pregnancy. Ensuring that patients with SLE are referred early during pregnancy, or ideally prior to conception, to a rheumatologist may improve their clinical outcomes. In addition, this study provides insight into outcomes in a population dissimilar to those previously studied. As more patients with SLE who are treated with HCQ complete pregnancy, larger studies will further elucidate the impact of HCQ on adverse maternal and neonatal outcomes. Expanding this study to other centers across the United States, as well as assessing the duration of SLE and HCQ use prior to pregnancy, would be important in future studies.

## Notes

This article meets the Accreditation Council for Graduate Medical Education and the American Board of Medical Specialties Maintenance of Certification competencies for Patient Care, Medical Knowledge, and Practice-Based Learning and Improvement.
